# Phyto-Mediated Synthesis of Silver Nanoparticles Using *Terminalia chebula* Fruit Extract and Evaluation of Its Cytotoxic and Antimicrobial Potential

**DOI:** 10.3390/molecules25215042

**Published:** 2020-10-30

**Authors:** Veena Malligere Ankegowda, Shiva Prasad Kollur, Shashanka K. Prasad, Sushma Pradeep, Chandan Dhramashekara, Anisha S. Jain, Ashwini Prasad, Chandrashekar Srinivasa, Poojitha B. Sridhara Setty, S. M. Gopinath, Rajendra Prasad S., Ali H. Bahkali, Asad Syed, Chandan Shivamallu

**Affiliations:** 1 Department of Chemistry, Bangalore Institute of Technology, K.R. Road, V.V. Puram, Karnataka, Bangalore 560 004, India; veenamdy12@gmail.com; 2 Department of Sciences, Amrita School of Arts and Sciences, Amrita Vishwa Vidyapeetham, Mysuru Campus, Karnataka 570 026, India; 3 Department of Biotechnology and Bioinformatics, School of Life Sciences, JSS Academy of Higher Education and Research, Mysuru, Karnataka 570 015, India; shashankaprasad@jssuni.edu.in (S.K.P.); sushmap@jssuni.edu.in (S.P.); chandand@jssuni.edu.in (C.D.); 4 Department of Microbiology and Tissue Culture, School of Life Sciences, JSS Academy of Higher Education and Research, Mysuru, Karnataka 570 015, India; anishasjain@jssuni.edu.in (A.S.J.);; 5 Department of Biotechnology, Davangere University, Shivagangotri, Davangere, Karnataka 577 007, India; chandru.s@davangereuniversity.ac.in (C.S.); bspujitha@gmail.com (P.B.S.S.); gopinath@davangereuniversity.ac.in (S.M.G.); 6 Department of Chemistry, Davangere University, Shivagangotri, Davangere, Karnataka 577 007, India; raju.rajendraprasad693@gmail.com; 7 Department of Botany and Microbiology, College of Science, King Saud University, P.O. Box 2455, Riyadh 11451, Saudi Arabia; abahkali@ksu.edu.sa

**Keywords:** *T. chebula*, AgNPs, TEM, cytotoxicity, antibacterial activity

## Abstract

The increasing interest in developing potent non-toxic drugs in medicine is widening the opportunities for studying the usage of nanostructures in the treatment of various diseases. The present work reports a method for a facile and an eco-friendly synthesis of silver nanoparticles (AgNPs) using *Terminalia chebula* fruit extract (TCE). The obtained AgNPs was characterized by using different spectroscopic and microscopic techniques. The analysis of the results revealed that the as-obtained AgNPs have spherical morphology with an average diameter of 22 nm. Furthermore, the preliminary bioactivity evaluations revealed that the bio-conjugation of AgNPs, using TCE, significantly enhanced the antibacterial and anti-breast cancer potentials of the latter. The antibacterial activity of the as-prepared AgNPs showed that *B. subtilis* was more sensitive towards the AgNPs, followed by *P. aeruginosa*; while, *E. coli* and *S. mutans* showed comparatively minimal sensitivity toward the AgNPs. The IC_50_ values of TCE, AgNPs and TCE + AgNPs treatment of MCF-7 were found to be 17.53, 14.25 and 6.484 µg/mL, respectively. Therefore, it can be ascertained that the bio-conjugation may provide a headway with regard to the therapeutic employment of *T. chebula*, upon mechanistically understanding the basis of observed antibacterial and anticancer activities.

## 1. Introduction

The biosynthesis of nanoparticles has gained significant interest the field of biotechnology and nanotechnology with focused attention due to its proliferating need in the material synthesis. Prominent efforts have been made in the synthesis of metal nanoparticles using plants and microorganisms. Silver nanoparticles are profoundly important, and used as antimicrobial agents [1], in water purification systems [2] and in cosmetics [3]. Moreover, AgNPs are very much suitable for biological imaging and sensing as they exhibit strong optical properties [4]. Further, the AgNPs obtained using plants or plant extracts, using biological approaches, have become ecofriendly alternative tools against physical and chemical methods, thereby making them safer, and are preferred for human therapeutic use [5,6].

*Terminalia chebula* is the most widely used herb and commonly consumed traditional medicine of India [7]. It is well-documented traditional medicine to treat several diseases and infections [8]. *T. chebula* belongs to Combretaceae family and is found all over India and its fruit is considered as the “King of medicines”. This herb has achieved considerable development for its medicinal applications concerning several biological activities. Based on its unique natural components and products, it has been considered as a valuable source in developing medicines against infections and diseases [9]. *T. chebula* fruit is rich in phytochemical constituents like sterols, resin, amino acids, flavonoids, tannins etc., its content varies depending upon the geographical variation [10]. This plant is rich in tannins and the major chief polyphenol components of tannins are corialgin, chebulic acid, ellagic acid and gallic acid [11]. Further, the fruit extract of *T. chebula* has a antimicrobial activity as it was found to be very effective in both gram-negative and gram-positive bacteria, similar to *Staphylococcus aureus*, *Staphylococcus epidermidis*, *Bacillus subtilis* [12]. It also has an excellent antioxidant, anticarcinogenic, antidiabetic, antiaging activities [13]. *T. chebula* can also act as antiviral agent by activating the macrophage/monocyte of the system and also helps in the release TNF and interferon in the host cells [14]. In addition, *T. chebula* has been reported to be cytotoxic against cancer cell lines at higher concentrations. In a study conducted by Ravi Shankara et al. (2016), ethanolic extract of *T. chebula* leaf gall was found to effectively inhibit the growth of human lung cancer (A-549) and human breast adenocarcinoma (MCF-7) cell lines. The reported IC_50_ values against A-549 and MCF-7 were 208.16 ± 3.7, and 643.13 ± 4.2 µg/mL, respectively [15]. Furthermore, the aqueous extract of *T. chebula* was found to induce apoptosis in A-549 cell line via mitochondria-mediated pathway controlled by the Bcl-2 family members, Bcl-2, Bcl-xI and Bax proteins [16]. Similarly, in an in vivo study conducted by Ahuja et al. (2013), it was concluded that the ethanolic extract of *T. chebula* significantly reduced the tumor growth in mice, with EAC induced cancer, apart from increasing their lifespan and restoring the haematological homeostasis [17]. To the best of knowledge, limited research has been conducted in understanding the anti-breast cancer potential of *T. chebula* owing to its selective cytotoxicity.

AgNPs on the other hand are promising anticancer agents, which have an influence on the cell cycle and proliferation by the means of oxidative stress generation and apoptosis induction. Furthermore, they are antibacterial, anti-viral and anti-fungal [18]. Numerous studies have suggested that the used of plant-mediated silver nanoparticles show a greater cytotoxic effect against the cancer cell lines at lower-doses [19,20,21,22]. We have attempted to evaluate the plant-mediated AgNPs using the *T. chebula* extract for its anti-breast cancer potential. Therefore, considering the above facts on the medicinal importance of *T. chebula*, in this report, we have synthesized the silver nanoparticles (AgNPs) using *T. chebula* fruit extract, and characterized using XRD, TEM and HRTEM techniques, and studied its antimicrobial efficacy against *B. subtilis*, *E. coli*, *P. aeruginosa* and *S. Mutans* bacteria strains.

## 2. Materials and Methods

Precursors, silver nitrate obtained from S.D. Fine Chemicals Ltd. (Mumbai, India), methanol, ethanol and acetone were procured from Merck chemicals, Mumbai, India. DM water was collected from ELGA RO water purifier and used throughout the experiments (Elga Veolia, Lane End, UK). Powder XRD was measured using Bruker X-ray diffractometer using a Cu Kα (1.5406 Å) radiation (Bruker, Karlsruhe, Germany). Transmission electron microscopy (TEM) images and SAED pattern were recorded on JEOL 2100F FEG operating at 200 kV after casting a drop of AgNPs dispersion in ethanol over Cu grid (Jeol, Akishima, Tokyo, Japan).

### 2.1. Collection of T. chebula Fruit

The fruits of *T. chebula* were collected from surroundings of JSSAHER Mysuru campus, Karnataka. The selected dried fruits were washed with sterile distilled water to remove the surface adhered particles. The fruit material was authenticated by the Botanist. The fruits were then shade-dried in a dust free environment, the dried fruits were finely cut and then powdered for further analysis.

### 2.2. Preparation of T. chebula Fruit Extract

In order to prepare *T. chebula* fruit extract, 80 g of finely powdered sample is loaded into the thimble, a porous bag made from a strong filter paper or cellulose, which is placed on the thimble chamber of the Soxhlet apparatus. The extraction solvent, methanol is heated in the round bottom flask, which is attached to a Soxhlet extractor and condenser. Methanol vaporizes into the sample thimble, condenses in the condenser and drip back. Once the level of solvent reaches the siphon it pours back into the flask and the cycle begins again. This process continued for 48 h and finally the obtained methanolic extract was reduced under pressure.

### 2.3. Synthesis of AgNPs Using T. chebula Fruit Extract

In relation to the dried methanolic extract of *T. chebula* fruit, distilled water (5 mL) was added to get a concentration of 50 µg/mL. The above solution was transferred to an Erlenmeyer flask containing 0.1 mM AgNO_3_ (prepared using 25 mL deionised water) and the mixture was heated at 70 °C with constant stirring till the color change appears to brown. Moreover, similar results were observed upon repeating the experiments using aforementioned synthetic protocol. The solution was then cooled and allowed to halt at room temperature for 24 h followed by centrifugation at 15,000 rpm for 10 min. The supernatant obtained was discarded, and the pellet so formed was dispersed in 1 mL ethyl alcohol, followed by drying to get powdered AgNPs.

### 2.4. Antibacterial Potential Action of Biosynthesized AgNPs

The bactericidal activity tests were carried out with *Bacillus subtilis*, *Escherichia coli*, *Pseudomonas aeruginosa* and *S. mutans* bacteria in Nutrient Agar medium. Throughout this study, the same medium was used for all strains. The activity of Ag NPs towards all the bacterial cultures was done using the micro dilution method and well diffusion method.

### 2.5. Microdilution Method

The broth microdilution method was carried out in a 96-well microtiter plate to determine the minimum inhibitory concentration (MIC). The different concentrations of compounds (1, 0.5, 0.25 and 0.125 mg/mL) were diluted in Mueller Hinton broth and the final volume was maintained at 100 µL. The final concentration of DMSO was less than 1%. Five (5) µL of an overnight grown bacterial culture was added to the test medium to bring the final inoculum size to 1 × 10^5^ cfu/mL. The agar plates were incubated at 37 °C for 16 h and the absorbance was read at 600 nm. The percent growth inhibition was calculated by comparison with a control using the formula indicated below. The lowest concentration of the compound that inhibits the complete growth of the bacterium was determined as the MIC, mentioned in the below Formula (1):

% of growth inhibition = Control − Test/Control × 100.(1)

### 2.6. Evaluation of Zone of Inhibition (ZOI) by Well Diffusion Method

The bacterial lawn was prepared on sterile agar plates by using spread plate method. Cultures of *Bacillus subtilis, Escherichia coli, Pseudomonas aeruginosa* and *Streptococcus mutans* were used to make a lawn culture. Three wells of approximately 8 mm diameter were made. AgNPs of different concentrations were placed inside each well with a blank well with sterile water, including a streptomycin standard antibiotic. The plates were kept for incubation at 37 °C overnight. Zone diameter was measured. Values are expressed as mean ± SE. Statistical significance was determined using one-way analysis of variance (ANOVA) and values with *p* < 0.05 were considered significant.

### 2.7. Evaluation of In Vitro Anticancer Activity Assay

The anti-proliferative effects of TCE, AgNPs and TCE + AgNPs were determined, using the MTT assay [23] on breast cancer (MCF-7) cell line procured from National Centre for Cell Science (NCCS), Pune, India. The cells were cultured in Dulbecco’s Modified Eagle Medium (DMEM), containing 10% Fetal Bovine Serum (FBS), 100 IU/mL penicillin, and 100 µg/mL streptomycin, in 5% CO_2_, at 37 °C, until confluent. The cells were trypsinized with 0.05% trypsin-EDTA solution for the haemocytometric cellular viability screening before 10,000 cells/well were plated and incubated in 5% CO_2_ at 37 °C, until confluent. The treatment was carried out at the TCE, AgNPs and TCE + AgNPs concentrations of 2.5, 5, 10, 20 and 400 µg/mL. The cells were fixed using 5 mg/mL of MTT reagent per well, after 24 h of treatment, and incubated at 37 °C for 1 h, followed by centrifugation for 5 min at 3000 rpm. The plates were then wash with distilled water to remove the excess dye and air dried. 100 μL of DMSO was added on to the plate, to solubilize the formazan crystals formed, and OD was read at 570 nm. % inhibition was calculated using the below Formula (2):

(2) $$\%\;\mathrm{inhibition}=100-\left[\frac{\mathrm{OD}\;\mathrm{of}\;\mathrm{sample}-\mathrm{OD}\;\mathrm{of}\;\mathrm{blank}}{\mathrm{OD}\;\mathrm{ofcontrol}-\mathrm{OD}\;\mathrm{of}\;\mathrm{blank}}\right]\times 100$$

The % inhibition was represented graphically and statistically using the Prism 8 statistical analysis tool (GraphPad Software, San Diego, CA, USA).

## 3. Results and Discussion

The pure AgNPs is prepared using the reported method elsewhere [24]. However, the as-prepared AgNPs is synthesized using the fruit extract of *T. chebula* as detailed in Section 2.3.

### 3.1. Powder X-ray Diffractionm (XRD) Analysis

**Figure 1:**
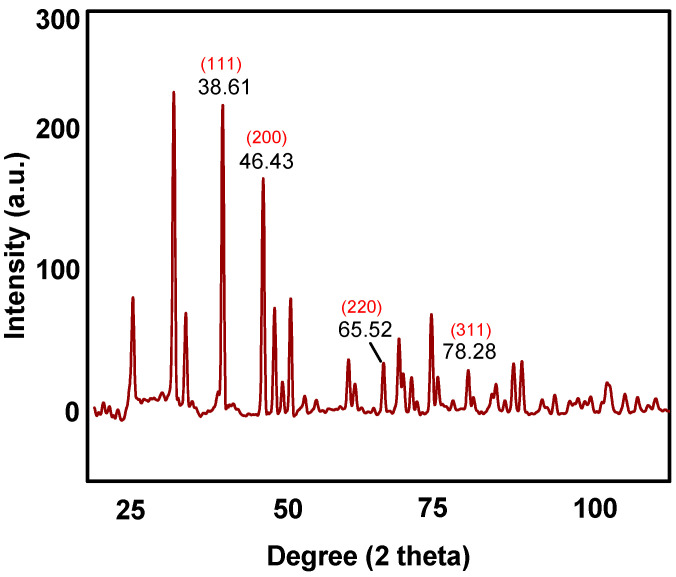
X-ray diffraction pattern of as-prepared AgNPs.

The XRD diffraction patterns of as-obtained AgNPs is manifested in Figure 1. The Braggs reflections noticed at 2θ = 38.61°, 46.43°, 65.52° and 78.28° correspond to (111), (200), (220) and (311) lattice planes, respectively which can be further attributed to the face centered cubic crystal structure of silver. Furthermore, the XRD pattern revealed the crystalline nature of as-obtained AgNPs.

### 3.2. Transmission Electron Microscopy Analysis

**Figure 2:**
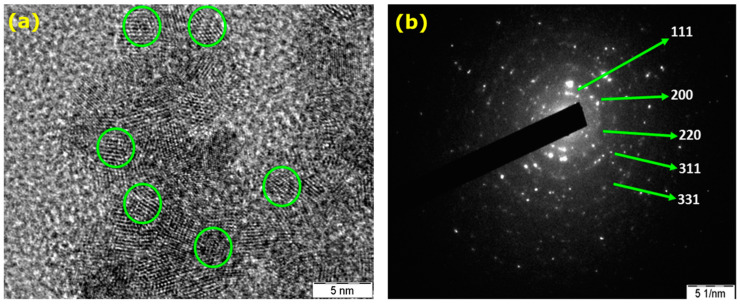
(**a**) HR-TEM and (**b**) SAED images of AgNPs prepared using *T. chebula* fruit extract.

The morphology and the size of the green synthesized AgNPs was investigated by High Resolution Transmission Electron Microscopy (HR-TEM) analysis. Although, previous reports mention the different morphology of AgNPs synthesized using a similar method [25], in our synthetic strategy, the as-prepared AgNPs is typically spherical in shape (Figure S1). The as-obtained AgNPs exhibited well-defined structures in nanoscale dimensions with typical spherical morphologies as depicted in Figure 2a, with an average particle size of 22 nm. As manifested in Figure 2a, the lattice fringes having inter planar distance of 0.31 nm, which is assigned to (111) plane. Furthermore, the crystallographic features of the as-prepared AgNPs was analyzed by Selected Area Electron Diffraction (SAED) studies. It is evident from SAED image (Figure 2b) that few spots of diffraction are spread in concentric circles, when the electron diffraction was performed on a limited number of crystals. The ring patterns observed for as-prepared AgNPs having plane distances of 2.39 Å, 2.07 Å, 1.45 Å, 1.25 Å, and 0.97 Å are in good agreement with the planes those could be indexed to (111), (200), (220), (311), and (331) of pristine face centered cubic silver structure (JCPDS file No. 4-0787). Moreover, these results are in consistent with the XRD pattern also.

### 3.3. Antibacterial Activity of AgNPs Prepared Using T.chebula Fruit Extract

**Figure 3:**
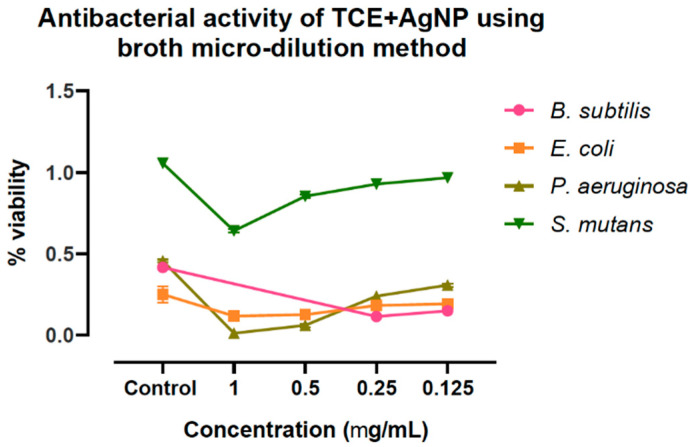
Antibacterial activity of AgNPs prepared using *T. chebula* fruit extract showing MIC.

The as-prepared AgNPs showed considerable dose-dependent antibacterial activity against the tested pathogenic bacterial strains. The measured zone of inhibition (Table S1; Figure S2) suggested that, *B. subtilis* was more sensitive to the AgNPs, followed by *P. aeruginosa*; while *E. coli* and *S. mutans* showed comparatively minimal sensitivity toward the AgNPs. This was also confirmed from the MIC of AgNPs measured against these bacteria (Figure 3). *B. subtilis* showed the least MIC than others. This could be explained by higher affinity of *B. subtilis* cells to colloidal AgNPs than the other tested bacterial strains [26]. These results showed that as-prepared AgNPs has significant effect on growth inhibition of Gram-negative bacteria than that of Gram-positive bacteria. This might be due to differences in the structure and composition of the cell wall of these bacteria [27]. Compared to the respective control (Table S2), the order of the observed antibacterial activity could be listed as *B. subtilis* > *P. aeruginosa* > *E. coli* > *S. mutans*. Antibacterial activity occurs in two steps; firstly phytochemicals adhered on the surface of AgNPs cause damage to the outer cell membrane by damaging the cell wall proteins which causes leakage of cellular matter. According to the widely accepted mechanism, the AgNPs enters into cell membrane, which rupture and the intracellular substances leak, which in turn affects the respiration process by inactivating the respiratory chain dehydrogenases and finally inhibits the respiration, as well as bacterial growth [28].

### 3.4. Cytotoxicity of Plant-Mediated AgNPs Using T. chebula

**Figure 4:**
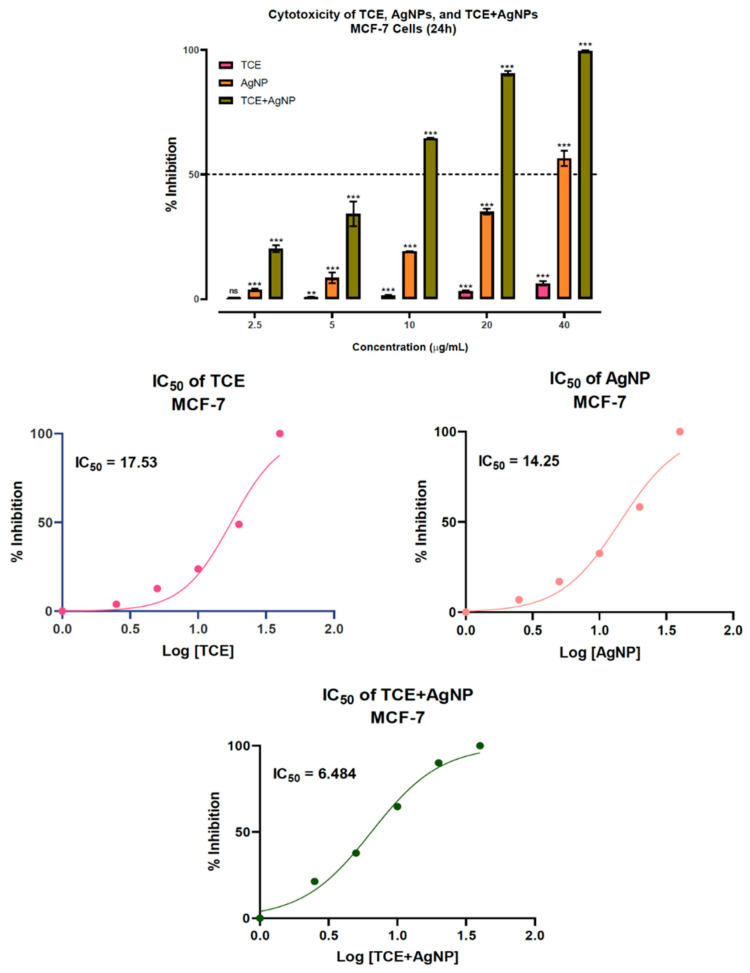
Cytotoxicity and IC_50_ values of *T. chebula* extract (TCE), silver nanoparticles (AgNPs), and TCE-mediated silver nanoparticles (TCE + AgNPs) treatment on human breast adenocarcinoma (MCF-7) cells. ** *p* = <0.002, *** *p* = <0.001.

TCE was found to be cytotoxic to the MCF-7 in a dose-dependent fashion. Notwithstanding this, high concentrations of TCE were required to show a significance in the observed anti-proliferative effect, compared to the AgNPs. However, the TCE-mediated preparation of AgNPs drastically enhanced the tumoricidal potential of the constituents even at very low concentrations. The IC_50_ values of 17.53 and 14.25 µg/mL were recorded for the separated treatments of MCF-7 with TCE and AgNPs (Figure 4). Surprisingly, significant reduction of the IC_50_ to 6.484 µg/mL, which is almost one third of TCE and half of AgNPs treatments, was observed in the TCE + AgNPs treatment of MCF-7 cells. Suggesting that the preparation of AgNPs using TCE enhanced the cytotoxic behavior. Similar observations were made by Satpathy et al. (2018), where the green synthesis of AgNPs using *P. tuberosa* extracts enhanced the cytotoxicity against breast, ovarian, multidrug resistant and brain cancer cell lines [29]. Patra et al. (2018) endorsed and suggested such improvement in biological activities in the medicinal plants when in combination with a metal nanoparticle. In this study, the bio-conjugation of AgNPs with *Saraca asoca* not just improved the anticancer, but also the antibacterial, antidiabetic as well as catalytic activities of the plant extract [30].

## 4. Conclusions

In summary, we have synthesized AgNPs using a medicinally important plant, *T. chebula* (fruit extract). The spherical morphology and the size of as-obtained AgNPs was determined by HRTEM analysis. Further, we have studied the preliminary potency of the as-obtained AgNPs as antibacterial and tumoricidal agents. It is noteworthy that the bio-conjugation of AgNPs using TCE was found to significantly enhance the bioactivities. Furthermore, it may be concluded that the bio-conjugation of AgNPs provide an ample scope for therapeutic usage of *T. chebula*, provided adequate mechanistic evaluations are carried out.

## Supplementary Materials

Figure S1: TEM image depicting the spherical nature of as-prepared AgNPs. Table S1: Antibacterial activity of AgNPs prepared using *T. chebula* fruit extract. Table S2: Antibacterial activity of silver nanoparticles without organic components prepared using *T. chebula* fruit extract.

## References

[1] Shruthi G., Prasad K.S., Vinod T.P., Balamurugan V., Shivamallu C. (2017). Green Synthesis of Biologically Active Silver Nanoparticles through a Phyto-Mediated Approach Using Areca Catechu Leaf Extract. ChemistrySelect.

[2] Yaqoob A.A., Parveen T., Umar K., Ibrahim M.N.M. (2020). Role of Nanomaterials in the Treatment of Wastewater: A Review. Water.

[3] Huq A. (2020). Green Synthesis of Silver Nanoparticles Using Pseudoduganella eburnea MAHUQ-39 and Their Antimicrobial Mechanisms Investigation against Drug Resistant Human Pathogens. Int. J. Mol. Sci..

[4] Kollur S.P., Shruthi G., Shivamallu C. (2018). Functionalized Silver Nano-Sensor for Colorimetric Detection of Hg2^+^ Ions: Facile Synthesis and Docking Studies. Sensors.

[5] Burdușel A.-C., Gherasim O., Grumezescu A.M., Mogoantă L., Ficai A., Andronescu E. (2018). Biomedical Applications of Silver Nanoparticles: An Up-to-Date Overview. Nanomaterials.

[6] Hamouda R.A., Hussein M.H., Abo-Elmagd R.A., Bawazir S.S. (2019). Synthesis and Biological Characterization of Silver Nanoparticles Derived from the Cyanobacterium Oscillatoria Limnetica. Sci. Rep..

[7] Assie J., Fatemeh M., Omid S., Mohsen N.T., Shokouhsadat H. (2016). Potential Therapeutic Applications for Terminalia Chebula in Iranian Traditional Medicine. J. Tradit. Chin. Med..

[8] Rao N.K., Nammi S. (2006). Antidiabetic and Renoprotective Effects of the Chloroform Extract of Terminalia Chebula Retz. Seeds in Streptozotocin-Induced Diabetic Rats. BMC Complement. Altern. Med..

[9] Srinivas T.L., Lakshmi S.M., Shama S.N. (2013). Medicinal Plants as Anti-Ulcer Agents. J Pharmacogn. Phytochem..

[10] Kaur S., Jaggi R.K. (2010). Antinociceptive Activity of Chronic Administration of Different Extracts of Terminalia Bellerica Roxb. and Terminalia Chebula Retz. Fruits. Indian J. Exp. Biol..

[11] Fraga-Corral M., García-Oliveira P., Pereira A.G., Lourenço-Lopes C., Jimenez-Lopez C., Prieto M., Simal-Gandara J. (2020). Technological Application of Tannin-Based Extracts. Molecules.

[12] Bag A., Bhattacharyya S.K., Bharati P. (2009). Evaluation of Antibacterial Properties of Chebulic Myrobalan (Fruit of Terminalia Chebula Retz.) Extracts Against Methicillin Resistant Staphylococcus Aureus and Trimethoprim-Sulpha-Methoxazole Resistant Uropathogenic Escherichia Coli. Afr. J. Plant Sci..

[13] Lee H.-S., Jung S.-H., Yun B.-S., Lee K.-W. (2006). Isolation of Chebulic Acid from Terminalia Chebula Retz. and Its Antioxidant Effect in Isolated Rat Hepatocytes. Arch. Toxicol..

[14] Trinh T.A., Park J., Oh J.H., Park J.S., Lee D., Kim C.-E., Choi H.-S., Kim S.-B., Hwang G.S., Koo B.A. (2020). Effect of Herbal Formulation on Immune Response Enhancement in RAW 264.7 Macrophages. Biomolecules.

[15] Shankara B.E.R., Ramachandra Y.L., Rajan S.S., Ganapathy P.S.S., Yarla N.S., Richard S.A., Dhananjaya B.L. (2016). Evaluating the Anticancer Potential of Ethanolic Gall Extract of Terminalia Chebula (Gaertn.) Retz. (Combretaceae). Pharmacogn. Res..

[16] Wang M., Yang L., Ji M., Zhao P., Sun P., Bai R., Tian Y., Su L., Li C. (2015). Aqueous Extract of Terminalia Chebula Induces Apoptosis in Lung Cancer Cells Via a Mechanism Involving Mitochondria-mediated Pathways. Braz. Arch. Biol. Technol..

[17] Ahuja R., Agrawal N., Mukerjee A. (2013). Evaluation of Anticancer Potential of Terminalia Chebula Fruits Against Ehrlich Ascites Carcinoma Induced Cancer in Mice. J. Sci. Innov. Res..

[18] Skonieczna M., Hudy D. (2018). Biological Activity of Silver Nanoparticles and Their Applications in Anticancer Therapy. Silver Nanoparticles Fabr. Charact. Appl..

[19] Pei J., Fu B., Jiang L., Sun T. (2019). Biosynthesis, Characterization, and Anticancer Effect of Plant-Mediated Silver Nanoparticles Using Coptis Chinensis. Int. J. Nanomed..

[20] Khan R.A., Tăbăcaru A., Ali F., Koo B.H. (2019). Anticancer and Antimicrobial Properties of Inorganic Compounds/Nanomaterials. Bioinorg. Chem. Appl..

[21] Madhu C., Balaji K., Sharada A., Shankar J. (2017). Anticancer Effect of Silver Nanoparticles (AgNP’s) from Decalepis Hamiltonii: An In Vivo Approach. Mater. Today Proc..

[22] Prasad K.S., Prasad S.K., Ansari M.A., Alzohairy M.A., Alomary M.N., Alyahya S., Srinivasa C., Murali M., Ankegowda V.M., Shivamallu C. (2020). Tumoricidal and Bactericidal Properties of ZnONPs Synthesized Using *Cassia auriculata* Leaf Extract. Biomolecules.

[23] Denizot F., Lang R. (1986). Rapid Colorimetric Assay for Cell Growth and Survival. Modifications to the Tetrazolium Dye Procedure Giving Improved Sensitivity and Reliability. J. Immunol. Methods.

[24] Pal A., Shah S., Devi S. (2009). Microwave-Assisted Synthesis of Silver Nanoparticles Using Ethanol as a Reducing Agent. Mater. Chem. Phys..

[25] Kumar K.M., Sinha M., Mandal B.K., Ghosh A.R., Kumar K.S., Reddy P.S. (2012). Green Synthesis of Silver Nanoparticles Using Terminalia Chebula Extract at Room Temperature and Their Antimicrobial Studies. Spectrochim. Acta Part A Mol. Biomol. Spectrosc..

[26] Bondarenko O., Ivask A., Käkinen A., Kurvet I., Kahru A. (2013). Particle-Cell Contact Enhances Antibacterial Activity of Silver Nanoparticles. PLoS ONE.

[27] Fayaz M., Balaji K., Girilal M., Yadav R., Kalaichelvan P.T., Venketesan R. (2010). Biogenic Synthesis of Silver Nanoparticles and Its Synergetic Effect with Antibiotics: A Study Against Gram Positive and Gram Negative Bacteria. Nanomedicine.

[28] Sondi I., Salopek-Sondi B. (2004). Silver Nanoparticles as Antimicrobial Agent: A Case Study on *E. Coli* as a Model for Gram-Negative Bacteria. J. Colloid Interface Sci..

[29] Satpathy S., Patra A., Ahirwar B., Hussain M.D. (2018). Antioxidant and Anticancer Activities of Green Synthesized Silver Nanoparticles Using Aqueous Extract of Tubers of Pueraria Tuberosa. Artif. Cells Nanomed. Biotechnol..

[30] Patra N., Kar D., Pal A., Behera A. (2018). Antibacterial, Anticancer, Anti-Diabetic and Catalytic Activity of Bio-Conjugated Metal Nanoparticles. Adv. Nat. Sci. Nanosci. Nanotechnol..

