# Supplementary material for: Phyto-Mediated Synthesis of Silver Nanoparticles Using Terminalia chebula Fruit Extract and Evaluation of Its Cytotoxic and Antimicrobial Potential
Source: Molecules. 2020 Oct 30;25(21):5042. doi: 10.3390/molecules25215042 (PMC7662631; doi:10.3390/molecules25215042)
Supplement: Supplementary file 1 [file molecules-25-05042-s001.pdf]

Supplementary material

The comparative study was carried out by taking pure AgNPs without any organic component. The bactericidal activity tests were carried out with *Bacillus subtilis, Escherichia coli, Pseudomonas aeruginosa* and *Streptococcus Mutans* bacteria in Nutrient Agar medium. The activity of silver nanoparticles without the organic component towards all the bacterial cultures was done using the well diffusion method.

Table S1: Antibacterial activity of silver nanoparticles without organic components prepared using *T. chebula* fruit extract.

**Tested bacteria against silver nanoparticles without organic components Diameter of inhibition zone standard (mm)**			
Bacillus subtilis	***Escherichia coli***	***Pseudomonas aeruginosa***	***Streptococcus Mutans***
6	4	5	6

Note: The experiments were conducted in triplicates.

© 2020 by the authors. Licensee MDPI, Basel, Switzerland. This article is an open access article distributed under the terms and conditions of the Creative Commons Attribution (CC BY) license (http://creativecommons.org/licenses/by/4.0/).
